# CoVeriTest with Adaptive Time Scheduling (Competition Contribution)

**DOI:** 10.1007/978-3-030-71500-7_18

**Published:** 2021-02-24

**Authors:** Marie-Christine Jakobs, Cedric Richter

**Affiliations:** 8 Universidad Autónoma de Madrid, Madrid, Spain; 9 University of Twente, Enschede, The Netherlands; 10 Department of Computer Science, Technical University of Darmstadt, Darmstadt, Germany; 11 Paderborn University, Paderborn, Germany

**Keywords:** Test-case generation, Cooperative Verification, CPAchecker

## Abstract

CoVeriTest, which is integrated in the analysis framework CPAchecker, adopts verification technology for test-case generation. It encodes individual test goals as reachability queries, which are then processed by verifiers. To increase the effectiveness on a broad class of testing tasks, CoVeriTest leverages the strengths of two different analyses: an explicit value analysis and predicate abstraction. Similar to TestComp’20, the two analyses are interleaved and the time duration of an interleaving segment is calculated dynamically. However, the calculation of the time duration focuses on the predicted future performance instead of the past performance, thus, rewarding analyses that likely cover open test goals.

## 1 Test-Generation Approach

Generating test-cases for a diverse set of tasks like in TestComp is challenging and often cannot be performed effectively by a single approach. Therefore, cooperative approaches that combine the strengths of multiple test-case generators frequently show superior performance as long as they do not spend too much time in unproductive test-case generators. To avoid unproductive test-case generation, we equip our CoVeriTest submission with a novel learning-based scheduler that considers the expected productiveness of a test-case generator.

CoVeriTest is a hybrid approach based on the concept of cooperative, verification-based testing [5], which combines complementary verifiers. In our current instantiation, we iteratively run two verification algorithms, namely value analysis [4] and predicate analysis [3]. In each iteration, the analyses proceed their exploration until they hit their time limit. The time limit of an analysis is computed dynamically at the beginning of each iteration round using our novel learning-based time scheduler. To generate test cases, we encode the (open) test

* This work was funded by the Hessian LOEWE initiative within the Software-Factory 4.0 project and it was partially supported by the German Research Foundation (DFG) within the Collaborative Research Centre "On-The-Fly Computing" (SFB 901) (grant number 160364472).

** jury-member

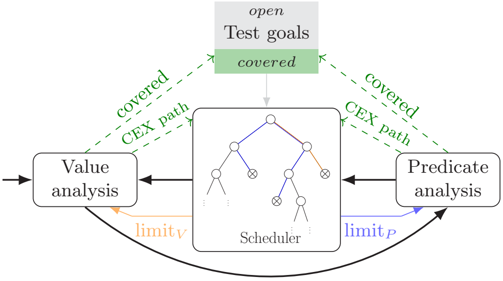

**Fig. 1.** Our adaptive scheduler integrated in the workflow of CoVeriTest goals, which are shared between the analyses, as unreachability queries and let the analyses prove the unreachability of those goals. A reported counterexample proves the reachability of a test goal. Therefore, the counterexample is converted into a test [1] and the test goal is removed from the set of open test goals.

**Time Scheduling.** Our time scheduler limits the time per iteration round to 100 s[3] and distributes the 100 s based on the expected contribution of the individual analyses. The idea is that an analysis gets more time if there exists more paths to open test goals that the analysis is expected to handle well.

Figure 1 shows the integration of our time scheduler into the CoVeriTest workflow. First, the scheduler samples a set of syntactical counterexample paths $\rho$, which starts at the beginning of the program and ends in an open test goal. Then, it estimates for each path $\rho$ the probability $P(V_i \mid \rho)$ that analysis $i$ detects $\rho$ as a real counterexample[4]. We estimate the probability $P(V_i \mid \rho)$ using an unigram language model [9] in combination with the approach of Richter et al. [10] for the abstraction of the syntactical paths $\rho$. Finally, the scheduler assigns a time budget to analysis $i$ in proportion to the average probability of detecting a counterexample path on a testing task $T$ (program plus open test goals):

$$\text{limit}_i^{\text{new}} = 10s + 80s * \mathbb{E}_{\rho \in T}[P(V_i \mid \rho)] \tag{1}$$

**Learning Probability Distribution.** The probability distribution $P(V_i \mid \rho)$ is unknown. Thus, we aim to learn the distribution. To this end, we executed the value and predicate analysis separately on the TestComp'20 category coverage-branches and used the reported counterexamples, which are obviously counter-examples that can be decided by the reporting analysis, to pre-train our unigram language model [9]. At the beginning of each CoVeriTest execution, we load the pre-trained model and use the reported counterexamples to improve it during

[3] We choose the same iteration time limit as in TestComp'20 [8], which has been established by extensive evaluation of CoVeriTest [5].

[4] Note that it is not important that $\rho$ is a real counterexample. We rather model the probability that the analysis $i$ can decide whether $\rho$ is a counterexample than to decide whether $\rho$ is a counterexample.

execution. When the sampled paths are indecisive, $\mathbb{E}_{\rho \in T}[P(V_i \mid \rho)]$ becomes the normalized progress used in the TestComp'20 strategy [8]. The normalized progress describes the relative contribution of an analysis to the goals covered in the last iteration.

## 2 Tool Architecture

CoVeriTest is an extension of the software analysis framework CPAchecker [2] (version 2.0) and is written in Java. For parsing, we use the Eclipse CDT parser[5]. For test-case generation, we rely on two instances of CPAchecker's test-case generation algorithm, which extracts test cases from counterexamples [1]. One instance generates test cases based on CPAchecker's value analysis [4] and the other instance uses CPAchecker's predicate analysis [3]. Both analyses apply counterexample-guided abstraction refinement [7] and use the SMT solver MathSAT5 [6]. We interleave the two instances and determine their time slices based on their expected success on the set of open test goals. To determine the time slices, we added the adaptive scheduler described in the previous section.

## 3 Strengths and Weaknesses

The main difference between CoVeriTest versions in Test-Comp'20 and Test-Comp'21 is the distribution of the 100 s per round. Our own experiments with the Test-Comp 2020 benchmark set revealed a small advantage for our new distribution with respect to the coverage-branches category. Comparing the competition results against a CoVeriTest configuration using the time distribution from Test-Comp'20 shows that the new distribution performs slightly worse in the `coverage-error` category. In total, 13 errors are missed, 8 of them are missed in the subcategory `Floats`. Overall, an advantage of the new distribution is scarcely noticeable on the Test-Comp 2021 benchmark set. The unigram language model does not generalize well.

Since the underlying analyses remain the same, CoVeriTest still generates a small number of test cases. Also, the problems with tasks using large arrays and the subcategories `BusyBox-Memsafety` and `SQLite-Memsafety` remain. Additionally, CoVeriTest performs poorly on the new `ntdrivers` tasks and the new subcategory `Combinations`. While finding the error in the new `nla-digbench` tasks is difficult, covering branches works well for these tasks. Moreover, CoVeriTest deals well with the new category `XCSP` and the remaining new tasks.

## 4 Setup

We develop our extension of CoVeriTest in a fork[6] of CPAchecker and submitted revision `970d550`, which participated in all categories. To run CoVeriTest

[5] https://www.eclipse.org/cdt/
[6] https://github.com/cedricrupb/cpachecker on program `program.i`, one requires a Java 11 runtime environment and must execute the following command line:

```
scripts/cpa.sh -testcomp21 -setprop log.consoleLevel=SEVERE -stats
   -benchmark -heap 10000m -spec property.prp program.i
```

Note that `property.prp` is a place marker for the test specification (`coverage--error-call.prp` or `coverage-branches.prp`). Tests are generated for programs assuming a 32-bit environment. To support 64-bit environments, one needs to add the configuration option -64. The generated tests are written to the folder `output/test-suite` and adhere to the XML format demanded by the Test-Comp rules. Additionally, the folder contains the mandatory metadata file.

## 5 Project and Contributors

CoVeriTest is an extension of the CPAchecker project[7] and is developed as a joint, open source project between research groups of Paderborn University and TU Darmstadt. Contributors are Marie-Christine Jakobs and Cedric Richter. We also like to thank all developers of CPAchecker.

## References

[1] Beyer, D., Chlipala, A.J., Henzinger, T.A., Jhala, R., Majumdar, R.: Generating tests from counterexamples. In: Proc. ICSE. pp. 326–335. IEEE (2004), 10.1109/ICSE.2004.1317455

[2] Beyer, D., Keremoglu, M.E.: CPAchecker: A tool for configurable software verification. In: Proc. CAV. pp. 184–190. LNCS 6806, Springer (2011), 10.1007/978-3-642-22110-1_16

[3] Beyer, D., Keremoglu, M.E., Wendler, P.: Predicate abstraction with adjustable-block encoding. In: Proc. FMCAD. pp. 189–197. FMCAD (2010), http://ieeexplore.ieee.org/document/5770949/

[4] Beyer, D., Löwe, S.: Explicit-state software model checking based on CEGAR and interpolation. In: Proc. FASE. pp. 146–162. LNCS 7793, Springer (2013), 10.1007/978-3-642-37057-1_11

[5] Beyer, D., Jakobs, M.: CoVeriTest: Cooperative verifier-based testing. In: Proc. FASE. pp. 389–408. LNCS 11424, Springer (2019), 10.1007/978-3-030-16722-6_23

[6] Cimatti, A., Griggio, A., Schaafsma, B.J., Sebastiani, R.: The MathSAT5 SMT solver. In: Proc. TACAS. pp. 93–107. LNCS 7795, Springer (2013), 10.1007/978-3-642-36742-7_7

[7] Clarke, E.M., Grumberg, O., Jha, S., Lu, Y., Veith, H.: Counterexample-guided abstraction refinement for symbolic model checking. J. ACM **50**(5), 752–794 (2003), 10.1145/876638.876643

[8] Jakobs, M.: CoVeriTest with dynamic partitioning of the iteration time limit (competition contribution). In: Proc. FASE. pp. 540–544. LNCS 12076, Springer (2020), 10.1007/978-3-030-45234-6_30

[9] Jurafsky, D.: Speech & language processing. Pearson Education India (2000)

[10] Richter, C., Hüllermeier, E., Jakobs, M.C., Wehrheim, H.: Algorithm selection for software validation based on graph kernels. JASE **27**(1), 153–186 (2020), 10.1007/s10515-020-00270-x

